# Supplementary material for: Telomere-to-Telomere Gap-Free Genome Assembly and Comparative Analysis of the Opsariichthys bidens (Cypriniformes: Xenocyprididae)
Source: Biology (Basel). 2025 Nov 3;14(11):1544. doi: 10.3390/biology14111544 (PMC12650118; doi:10.3390/biology14111544)
Supplement: Supplementary file 1 [file biology-14-01544-s001.zip › biology-3949818-supplementary.pdf]

## Supplementary Materials

### Figure Legends

**Supplementary Figure S1.** K-mer distribution of different copy numbers in the assembled sequence of *O. bidens* genome. (A). Short read length data. (B). Long read length data.

**Supplementary Figure S2.** Chromosome element density distribution map (using chr1-4 as examples)

**Supplementary Figure S3.** Comparison of gene length.

**Supplementary Figure S4.** Venn diagram of the InterPro/GO/KEGG_KO/Swissprot/NR annotation.

**Supplementary Figure S5.** Estimates of the timing of species divergence.

**Supplementary Figure S6.** Enrichment of expanded gene families of *O. bidens.*

**Supplementary Figure S7.** Enrichment of contracted gene families of *O. bidens.*

**Supplementary Figure S8.** Enrichment of positive selection analysis of *O. bidens.*

### Table Legends

**Supplementary TableS1.** Species information used in comparative genomic analyses

**Supplementary Table S2.** The preliminary genome assembly results of *O. bidens* genome.

**Supplementary Table S3.** The Hi-C assisted assembly of *O. bidens* genome.

**Supplementary Table S4.** Statistics of reads alignment for *O. bidens* genome.reads

**Supplementary Table S5.** The consensus quality (QV) assessment of *O. bidens* genome.

**Supplementary Table S6**. The evaluation of genome assembly in *O. bidens.*

**Supplementary Table S7.** Homozygous and heterozygous rates of assembly for *O. bidens* genome.

**Supplementary Table S8.** Pathways were significantly enriched for PURs.

**Supplementary Table S9.** Statistics of repeated sequence classification for *O. bidens.*

**Supplementary Table S10**. The Repeat sequence statistics of *O. bidens* genome.

**Supplementary Table S11.** Gene prediction of assembly for *O. bidens* genome.

**Supplementary Table S12.** The Functional annotation of *O. bidens* genome.

**Supplementary Table S13.** Statistics of non-coding RNA annotation for *O. bidens* genome.

**Supplementary Table S14.** The evaluation of genome annotation in *O. bidens*

**Supplementary Table S15.** Statistics of the clustered gene families identified from the 12 fish species.

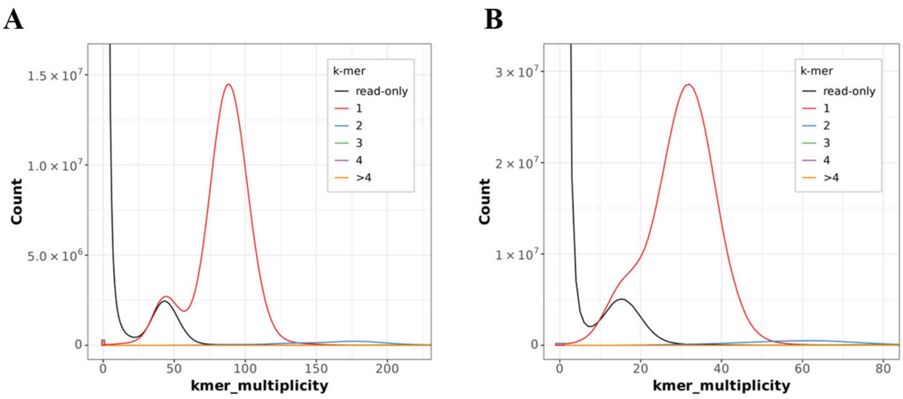


Figure S1 K-mer distribution of different copy numbers in the assembled sequence of *O. bidens* genome. A. Short read length data. B. Long read length data.

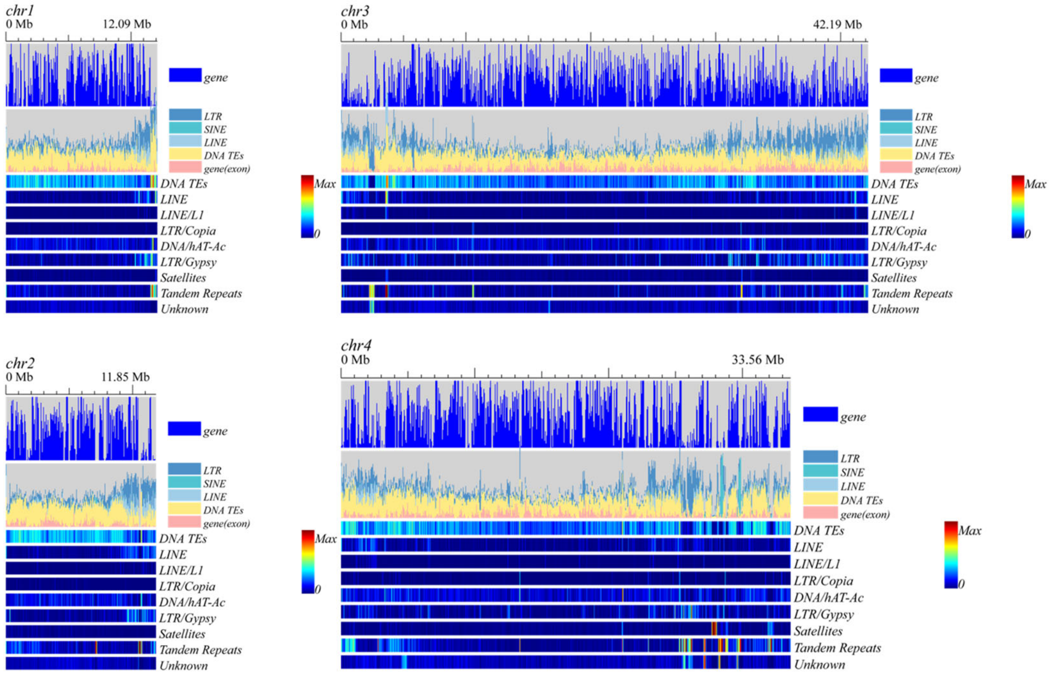


Figure S2 Chromosome element density distribution map (using chr1-4 as examples)

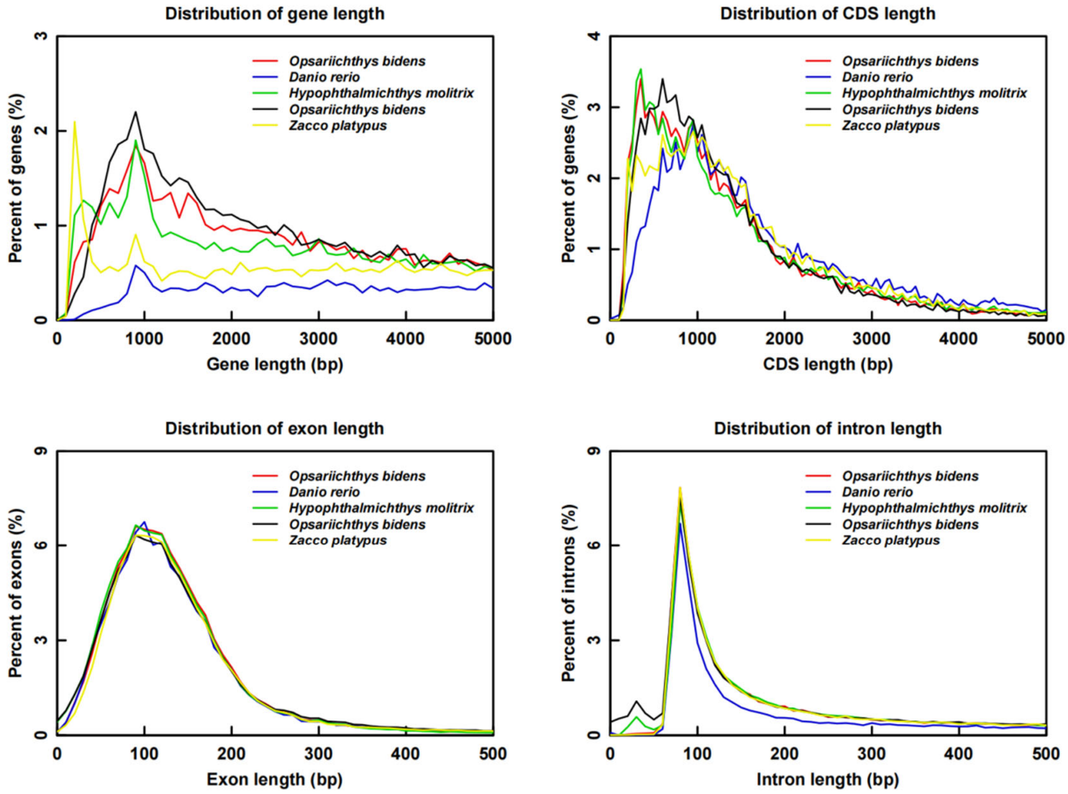


Figure S3 Comparison of gene length.

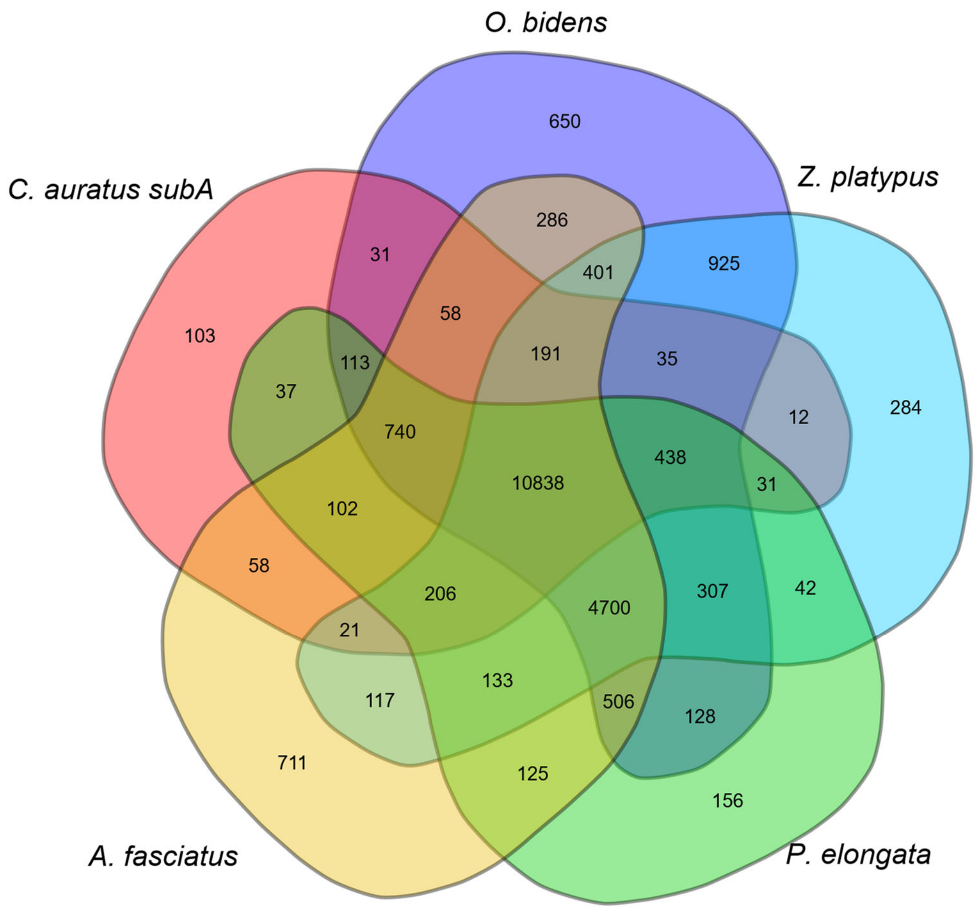


Figure S4. Venn diagram of the InterPro/GO/KEGG_KO/Swissprot/NR annotation.

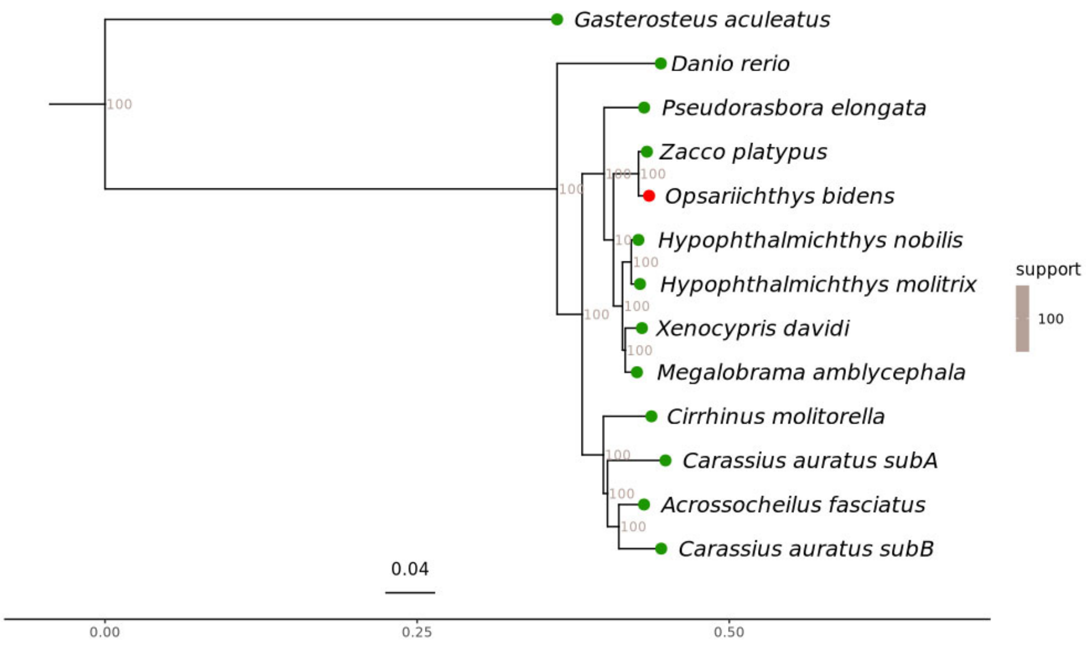


Figure S5 Phylogenetic tree of species.

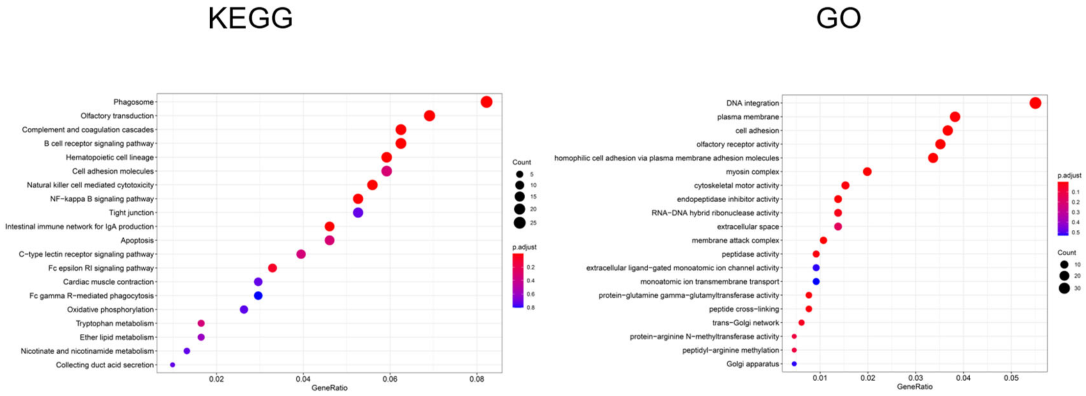


Figure S6.Enrichment of expanded gene families of *O. bidens*

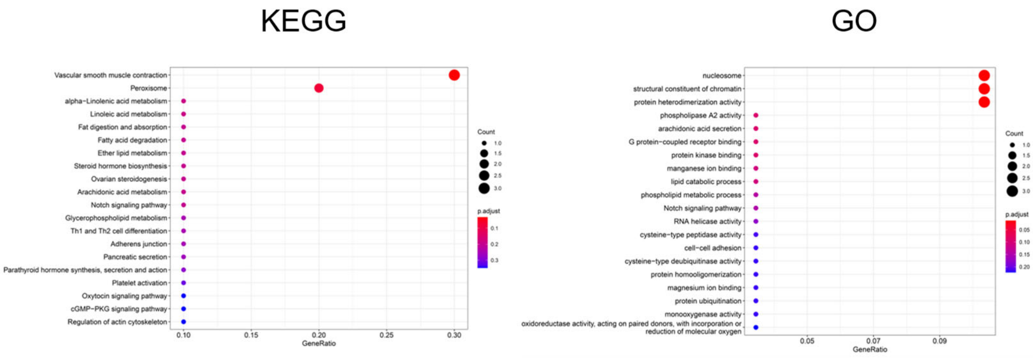


Figure S7.Enrichment of contracted gene families of *O. bidens*

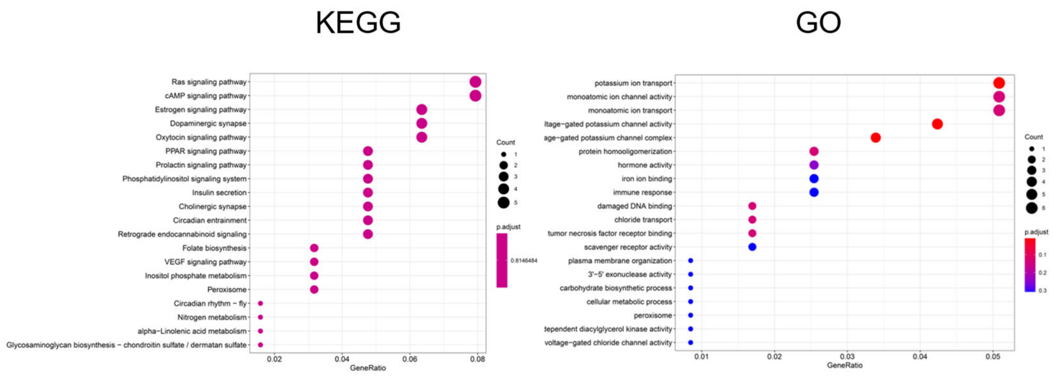


Figure S8. Enrichment of positive selection analysis of *O. bidens*.

TableS1. Species information used in comparative genomic analyses

| Species | Class | Order | Family | Genome links | Gff annotation links |
|---|---|---|---|---|---|
| *Opsariichthys bidens* | Cypriniformes | Cyprinidae | *Opsariichthys* | This study | |
| *Zacco platypus* | Cypriniformes | Cyprinidae | *Zacco* | GCA_034642465.1 | https://figshare.com/articles/dataset/Annotation_file_of_Zacco_platypus/24586665/1 |
| *Pseudorasbora elongata* | Cypriniformes | Cyprinidae | *Pseudorasbora* | /bigdata3/00.PM_result/OMTSEQ202404019_OMTBI-202412030026_Pseudorasbora_elongata_t2t | /bigdata3/00.PM_result/OMTSEQ202404019_OMTBI-202504070026_Pseudorasbora_elongata_anno |
| *Acrossocheilus fasciatus* | Cypriniformes | Cyprinidae | *Acrossocheilus* | GCA_036784905.1 | https://figshare.com/articles/dataset/Genome_annotations_of_i_Acrossocheilus_fasciatus_i_/24995825 |
| *Carassius auratus* | Cypriniformes | Cyprinidae | *Carassius* | GCA_003368295.1 | |
| *Cirrhinus molitorella* | Cypriniformes | Cyprinidae | *Cirrhinus* | GCA_033026305.1 | |
| *Danio rerio* | Cypriniformes | Cyprinidae | *Danio* | https://www.ncbi.nlm.nih.gov/datasets/genome/GCF_000002035.6/ | |
| *Gasterosteus aculeatus* | Gasterosteiformes | Gasterosteidae | *Gasterosteus* | https://www.ncbi.nlm.nih.gov/datasets/genome/GCF_016920845.1/ | |
| *Hypophthalmichthys molitrix* | Cypriniformes | Cyprinidae | *Hypophthalmichthys* | https://ftp.cngb.org/pub/CNSA/data3/CNP0000974/CNS0209133/CNA0019188/ | https://ftp.cngb.org/pub/CNSA/data3/CNP0000974/CNS0209133/CNA0019188/ |
| *Hypophthalmichthys nobilis* | Cypriniformes | Cyprinidae | *Hypophthalmichthys* | https://ftp.cngb.org/pub/CNSA/data3/CNP0000974/CNS0209134/CNA0019189/ | https://ftp.cngb.org/pub/CNSA/data3/CNP0000974/CNS0209134/CNA0019189/ |
| *Megalobrama amblycephala* | Cypriniformes | Cyprinidae | *Megalobrama* | GCA_018812025.1 | GCF_018812025.1 |
| *Xenocypris davidi* | Cypriniformes | Cyprinidae | *Xenocypris* | GCA_048182625.1 | https://figshare.com/articles/dataset/Genome_annotation_files_of_i_Xenocypris_davidi_i_/27932985 |

Table S2. The Preliminary genome assembly results of *O. bidens* genome.

| Mode | Total number | Total number (>= 2 kb) | Total length (bp) | max length (bp) | Mean length(bp) | N50 (bp) | N90 (bp) | GC content (%) |
|---|---|---|---|---|---|---|---|---|
| HiFi+Hi-C+ONT | 226 | 226 | 872,216,468 | 42,219,089 | 3,859,364 | 22,416,992 | 11,851,331 | 38.4 |

Table S3. The Hi-C assisted assembly of *O. bidens* genome.

| Genome version | Sequence Category | Scaffold | | | | Contig | | | |
|---|---|---|---|---|---|---|---|---|---|
| | | **Sequence length (bp)** | **Sequence number** | **N50 (bp)** | **N90 (bp)** | **Sequence length (bp)** | **Sequence number** | **N50 (bp)** | **N90 (bp)** |
| before anchored | Whole genome | 872,216,468 | 226 | 22,416,992 | 11,851,331 | 872,216,468 | 226 | 22,416,992 | 11,851,331 |
| after Anchored | Whole genome | 858,195,151 | 155 | 25,068,009 | 12,095,000 | 858,194,751 | 159 | 22,416,992 | 11,876,336 |
| | chromosome | 841,811,898 | 38 | 25,649,938 | 12,290,719 | 841,811,598 | 41 | 23,533,128 | 12,095,000 |
| | Free sequence | 16,383,253 | 117 | 531,646 | 48,320 | 16,383,153 | 118 | 531,646 | 48,305 |

Table S4. Statistics of reads alignment for *O. bidens* genome.reads

| **reads type** | **Mapping rate (%)** | **Paired mapping rate (%)** | **Coverage (%)** | **Coverage at least 4X (%)** | **Coverage at least 10X (%)** | **Coverage at least 20X (%)** |
|---|---|---|---|---|---|---|
| short reads | 99.15 | 91.71 | 99.94 | 99.85 | 99.71 | 99.45 |
| long reads | 99.72 | 32.12 | 99.82 | 98.81 | 89.60 | 99.72 |
| Nanopore ultra-long reads | 99.60 | 53.28 | 97.00 | 95.78 | 94.26 | 99.60 |

Table S5. The consensus quality (QV) assessment of *O. bidens* genome.

| **reads_type** | **K_asm** | **K_total** | **QV** | **Error rate** |
|---|---|---|---|---|
| short reads | 296,048 | 841,960,042 | 47.5489 | 1.75838e-05 |
| long reads | 212,954 | 841,960,042 | 48.9798 | 1.26478e-05 |

Table S6. The evaluation of genome assembly in *O. bidens*

| Software | Busco | | Compleasm | |
|---|---|---|---|---|
| Type | Proteins | Percentage (%) | Proteins | Percentage (%) |
| Complete BUSCOs | 3,616 | 99.34 | 3,634 | 99.84 |
| Complete Single-Copy BUSCOs | 3,596 | 98.79 | 3,626 | 99.62 |
| Complete Duplicated BUSCOs | 20 | 0.55 | 8 | 0.22 |
| Fragmented BUSCOs | 17 | 0.47 | 2 | 0.05 |
| Missing BUSCOs | 7 | 0.19 | 4 | 0.11 |
| Total BUSCO groups searched | 3,640 | 100.00 | 3,640 | 100.00 |
| Database | actinopterygii_odb10 | | actinopterygii_odb10 | |

Table S7. Homozygous and heterozygous rates of assembly for *O. bidens* genome.

| **Sample name** | **Homozygous SNP rate (%)** | **Homozygous InDel rate (%)** | **Heterozygous SNP rate (%)** | **Heterozygous InDel rate (%)** |
|---|---|---|---|---|
| *Opsariichthys_bidens* | 0.001 | 0.001 | 0.390 | 0.127 |

Table S8 Pathways were significantly enriched for PURs

| Enrichment | ID | Description | GeneRatio | BgRatio | pvalue | p.adjust | qvalue | Count |
|---|---|---|---|---|---|---|---|---|
| GO function | GO:0004497 | monooxygenase activity | 24/2686 | 82/17888 | 0.000721665142322431 | 0.0897634512924483 | 0.0897634512924483 | 24 |
| | GO:0016705 | oxidoreductase activity, acting on paired donors, with incorporation or reduction of molecular oxygen | 26/2686 | 92/17888 | 0.000795554912841966 | 0.0897634512924483 | 0.0897634512924483 | 26 |
| | GO:0042157 | lipoprotein metabolic process | 12/2686 | 31/17888 | 0.00110923804635097 | 0.0897634512924483 | 0.0897634512924483 | 12 |
| | GO:0004842 | ubiquitin-protein transferase activity | 36/2686 | 144/17888 | 0.00113864843077101 | 0.0897634512924483 | 0.0897634512924483 | 36 |
| | GO:0005506 | iron ion binding | 34/2686 | 136/17888 | 0.00153709111357948 | 0.103863442389014 | 0.103863442389014 | 34 |
| | GO:0004866 | endopeptidase inhibitor activity | 14/2686 | 44/17888 | 0.00386043114247883 | 0.200163395365325 | 0.200163395365325 | 14 |
| | GO:0005839 | proteasome core complex | 9/2686 | 23/17888 | 0.00423178425719503 | 0.200163395365325 | 0.200163395365325 | 9 |
| | GO:0051603 | proteolysis involved in protein catabolic process | 9/2686 | 23/17888 | 0.00423178425719503 | 0.200163395365325 | 0.200163395365325 | 9 |
| | GO:00 | heme | 27/26 | 120/1 | 0.018510572 | 0.79595461 | 0.79595461 | 27 |

| | | | | | | | | |
|---|---|---|---|---|---|---|---|---|
| | 20037 | binding | 86 | 7888 | 4452311 | 5144937 | 5144937 | |
| | GO:0005615 | extracellular space | 23/2686 | 100/17888 | 0.0220732804350133 | 0.870055137146774 | 0.870055137146774 | 23 |
| | GO:0005247 | voltage-gated chloride channel activity | 5/2686 | 12/17888 | 0.0239803452144213 | 0.872515637417021 | 0.872515637417021 | 5 |
| | GO:0006821 | chloride transport | 7/2686 | 21/17888 | 0.0288103391564236 | 0.973377887213455 | 0.973377887213455 | 7 |
| | GO:0006869 | lipid transport | 13/2686 | 51/17888 | 0.0350245160984715 | 0.999659157679084 | 0.999659157679084 | 13 |
| | GO:0005576 | extracellular region | 80/2686 | 440/17888 | 0.0372172393010215 | 0.999659157679084 | 0.999659157679084 | 80 |
| KEGG pathway | ko04621 | NOD-like receptor signaling pathway | 54/1222 | 249/8744 | 0.000516467323447734 | 0.119820419039874 | 0.119820419039874 | 54 |
| | ko04640 | Hematopoietic cell lineage | 28/1222 | 131/8744 | 0.0129399003741179 | 0.891864149382681 | 0.891864149382681 | 28 |
| | ko04745 | Phototransduction - fly | 12/1222 | 44/8744 | 0.0150068226359791 | 0.891864149382681 | 0.891864149382681 | 12 |
| | ko03020 | RNA polymerase | 9/1222 | 31/8744 | 0.0223134637423267 | 0.891864149382681 | 0.891864149382681 | 9 |
| | ko04514 | Cell adhesion molecules | 55/1222 | 304/8744 | 0.0242988345773831 | 0.891864149382681 | 0.891864149382681 | 55 |
| | ko04623 | Cytosolic DNA-sensing pathway | 13/1222 | 53/8744 | 0.0277064892895044 | 0.891864149382681 | 0.891864149382681 | 13 |
| | ko04662 | B cell receptor signaling pathway | 28/1222 | 141/8744 | 0.0324191531065764 | 0.891864149382681 | 0.891864149382681 | 28 |
| | ko04145 | Phagosome | 41/1222 | 223/8744 | 0.0374173194052689 | 0.891864149382681 | 0.891864149382681 | 41 |
| | ko04520 | Adherens junction | 28/1222 | 143/8744 | 0.0381529198619079 | 0.891864149382681 | 0.891864149382681 | 28 |

| | | | | | | | |
|---|---|---|---|---|---|---|---|
| ko04610 | Complement and coagulation cascades | 26/1222 | 131/8744 | 0.0384424202320121 | 0.891864149382681 | 0.891864149382681 | 26 |
| ko04612 | Antigen processing and presentation | 22/1222 | 109/8744 | 0.0456917472617319 | 0.963680487701982 | 0.963680487701982 | 22 |

Table S9. Statistics of repeated sequence classification for *O. bidens*

| | RepBase TEs | | TE Proteins | | *De novo* | | Combined TEs | |
|---|---|---|---|---|---|---|---|---|
| | Length (bp) | % in Genome | Length (bp) | % in Genome | Length (bp) | % in Genome | Length (bp) | % in Genome |
| DNA | 158,844,026 | 18.87 | 15,273,613 | 1.81 | 131,365,295 | 15.60 | 226,526,304 | 26.90 |
| LINE | 29,973,131 | 3.56 | 16,740,332 | 1.99 | 14,460,629 | 1.72 | 35,607,782 | 4.23 |
| SINE | 6,808,535 | 0.81 | 0 | 0.00 | 2,252,000 | 0.27 | 8,222,800 | 0.98 |
| LTR | 40,580,169 | 4.82 | 16,867,747 | 2.00 | 68,092,747 | 8.09 | 92,754,838 | 11.02 |
| Satellite | 12,343,178 | 1.47 | 0 | 0.00 | 3,010,807 | 0.36 | 14,609,971 | 1.74 |
| Simple_repeat | 0 | 0.00 | 0 | 0.00 | 7,432 | 0.00 | 7,432 | 0.00 |
| Other | 3,539 | 0.00 | 0 | 0.00 | 0 | 0.00 | 3,539 | 0.00 |
| Unknown | 4,079,941 | 0.48 | 5,346 | 0.00 | 39,276,353 | 4.66 | 42,304,429 | 5.02 |
| Total | 232,810,256 | 27.65 | 48,863,185 | 5.80 | 253,042,745 | 30.05 | 386,866,983 | 45.95 |

Table S10. The Repeat sequence statistics of *O. bidens* genome.

| **Type** | **Repeat Size (bp)** | **% of genome** |
|---|---|---|
| Trf | 86,572,594 | 10.28 |
| Repeatmasker | 232,810,256 | 27.65 |
| Proteinmask | 48,863,185 | 5.80 |
| De novo | 253,042,745 | 30.05 |
| Total | 407,457,363 | 48.39 |

Table S11. Gene prediction of assembly for *O. bidens* genome.

| **Gene set** | | **Protein coding gene number** | **Average gene length (bp)** | **Average CDS length (bp)** | **Average exon per gene** | **Average exon length (bp)** | **Average intron length (bp)** |
|---|---|---|---|---|---|---|---|
| De novo | Genscan | 29,492 | 18,943 | 1,603 | 8.44 | 189.98 | 2,332 |
| | AUGUSTUS | 36,737 | 11,651 | 1,304 | 7.23 | 180.35 | 1,660 |
| Homolog | *Opsariichthys bidens* | 40,715 | 10,173 | 1,443 | 7.11 | 202.99 | 1,430 |
| | *Zacco platypus* | 39,060 | 14,657 | 1,604 | 8.85 | 181.16 | 1,662 |
| | *Hypophthalmichthys molitrix* | 34,153 | 15,771 | 1,608 | 8.25 | 194.88 | 1,953 |
| | *Danio rerio* | 65,986 | 22,700 | 2,037 | 11.14 | 182.93 | 2,039 |
| Liftoff | *Danio rerio* | 22,181 | 13,376 | 1,222 | 5.84 | 209.13 | 2,510 |
| | *Hypophthalmichthys molitrix* | 26,720 | 13,202 | 1,475 | 8.28 | 178.19 | 1,611 |
| BUSCO | | 3,642 | 13,078 | 1,630 | 10.22 | 159.54 | 1,242 |
| MAKER | | 29,464 | 12,004 | 1,406 | 8.20 | 178.58 | 1,464 |
| HiFAP | | 29,816 | 13,047 | 1,553 | 8.42 | 187.49 | 1,547 |

Table S12. The Functional annotation of *O. bidens* genome

| | | Gene | | mRNA | |
|---|---|---|---|---|---|
| | | Number | Percent (%) | Number | Percent (%) |
| Total | | 29,816 | 100.00 | 29,816 | 100.00 |
| Annotated | | 27,169 | 91.12 | 27,169 | 91.12 |
| | NR | 27,061 | 90.76 | 27,061 | 90.76 |
| | SwissProt | 21,599 | 72.44 | 21,599 | 72.44 |
| | TrEMBL | 26,787 | 89.84 | 26,787 | 89.84 |
| | KOG | 19,924 | 66.82 | 19,924 | 66.82 |
| | TF | 6,440 | 21.60 | 6,440 | 21.60 |
| | InterPro | 24,071 | 80.73 | 24,071 | 80.73 |
| | GO | 17,888 | 59.99 | 17,888 | 59.99 |
| | KEGG_ALL | 25,893 | 86.84 | 25,893 | 86.84 |
| | KEGG_KO | 16,934 | 56.80 | 16,934 | 56.80 |
| | Pfam | 22,502 | 75.47 | 22,502 | 75.47 |
| Unannotated | | 2,647 | 8.88 | 2,647 | 8.88 |

Table S13. Statistics of non-coding RNA annotation for *O. bidens* genome

| Type | | Copy | Average length (bp) | Total length (bp) | Percentage of genome (%) |
|---|---|---|---|---|---|
| miRNA | | 1,165 | 88 | 102,048 | 0.012120 |
| tRNA | | 8,681 | 76 | 659,680 | 0.078350 |
| rRNA | rRNA | 22,396 | 119 | 2,656,844 | 0.315554 |
| | 18S | 0 | 0 | 0 | 0.000000 |
| | 28S | 0 | 0 | 0 | 0.000000 |
| | 5.8S | 18 | 154 | 2,763 | 0.000328 |
| | 5S | 22,378 | 119 | 2,654,081 | 0.315226 |
| snRNA | snRNA | 1,698 | 154 | 261,208 | 0.031024 |
| | CD-box | 268 | 155 | 41,527 | 0.004932 |
| | HACA-box | 73 | 153 | 11,141 | 0.001323 |
| | splicing | 1,347 | 153 | 206,182 | 0.024488 |
| | scaRNA | 10 | 236 | 2,358 | 0.000280 |

Table S14. The evaluation of genome annotation in *O. bidens*

| Software | Busco | | Compleasm | |
|---|---|---|---|---|
| Type | Proteins | Percentage (%) | Proteins | Percentage (%) |
| Complete BUSCOs | 3,542 | 97.31 | 3,545 | 97.39 |
| Complete Single-Copy BUSCOs | 3,485 | 95.74 | 3,012 | 82.75 |
| Complete Duplicated BUSCOs | 57 | 1.57 | 533 | 14.64 |
| Fragmented BUSCOs | 28 | 0.77 | 28 | 0.77 |
| Missing BUSCOs | 70 | 1.92 | 67 | 1.84 |
| Total BUSCO groups searched | 3,640 | 100.00 | 3,640 | 100.00 |
| Database | actinopterygii_odb10 | | actinopterygii_odb10 | |

Table S15. Statistics of the clustered gene families identified from the 12 fish species

| Species | Genes number | Unclustered genes | Genes in families | Family number | Unique families | Unique families genes | Common families | Common families genes | Single copy genes | Average genes per family |
|---|---|---|---|---|---|---|---|---|---|---|
| *O. bidens* | 29,816 | 2,149 | 27,667 | 20,347 | 69 | 307 | 6,433 | 9,137 | 2,933 | 1.360 |
| *Z. platypus* | 24,779 | 1,195 | 23,584 | 18,681 | 44 | 107 | 6,433 | 8,868 | 2,933 | 1.262 |
| *P. elongata* | 24,643 | 119 | 24,524 | 18,602 | 22 | 88 | 6,433 | 9,253 | 2,933 | 1.318 |
| *A. fasciatus* | 27,392 | 2,288 | 25,104 | 19,193 | 99 | 281 | 6,433 | 9,039 | 2,933 | 1.308 |
| *C. auratus subA* | 17,849 | 134 | 17,715 | 13,014 | 20 | 81 | 6,433 | 8,979 | 2,933 | 1.361 |
| *C. auratus subB* | 22,344 | 170 | 22,174 | 15,682 | 37 | 98 | 6,433 | 9,496 | 2,933 | 1.414 |
| *C. molitorella* | 25,865 | 1,716 | 24,149 | 18,655 | 74 | 474 | 6,433 | 8,980 | 2,933 | 1.295 |
| *D. rerio* | 26,453 | 271 | 26,182 | 18,252 | 86 | 709 | 6,433 | 10,105 | 2,933 | 1.434 |
| *G. aculeatus* | 22,186 | 1,241 | 20,945 | 16,001 | 196 | 870 | 6,433 | 9,055 | 2,933 | 1.309 |
| *H. molitrix* | 24,563 | 1,384 | 23,179 | 17,730 | 68 | 246 | 6,433 | 8,859 | 2,933 | 1.307 |
| *H. nobilis* | 24,225 | 757 | 23,468 | 17,481 | 27 | 67 | 6,433 | 8,958 | 2,933 | 1.342 |
| *M. amblycephala* | 29,735 | 95 | 29,640 | 19,119 | 33 | 86 | 6,433 | 10,409 | 2,933 | 1.550 |
| *X. davidi* | 26,574 | 1,141 | 25,433 | 18,894 | 99 | 323 | 6,433 | 8,782 | 2,933 | 1.346 |
